# The Relationship Between Functional Seizures, Dissociation, and Gender Dysphoria: A Case Report and Review

**DOI:** 10.7759/cureus.80267

**Published:** 2025-03-08

**Authors:** Brendan Coyne, Mariam Elghazzawy, Bo Ram Yoo, Varun S Mehta, Mahdieh Bodaghi

**Affiliations:** 1 Psychiatry, George Washington University School of Medicine and Health Sciences, Washington, USA; 2 Psychiatry, University of California (UC) San Diego, San Diego, USA; 3 Psychiatry, Icahn School of Medicine at Mount Sinai, New York City, USA; 4 Psychiatry and Behavioral Sciences, Children's National Hospital, Washington, USA

**Keywords:** dissociative disorder, functional neurologic disorder, functional seizure, lgbtq medicine, mood disorder, neuropsychiatric symptoms, psychogenic non-epileptic seizures (pnes), psychological trauma, suicide and depression, transgender and non-binary

## Abstract

Psychogenic non-epileptic seizures (PNES), also known as functional seizures, clinically mimic seizure-like activity without the presence of brain wave abnormalities on electroencephalogram (EEG). PNES, among other functional neurological disorders, demonstrates an increased prevalence among transgender and non-binary individuals, particularly those with ongoing gender dysphoria. It is established that PNES episodes can be triggered by states of severe distress and that mirror-gazing may cause distress in individuals with gender dysphoria. Herein, we review the available research on the pathophysiology of PNES. We also describe a 16-year-old non-binary patient with gender dysphoria, initially admitted following a suicide attempt, who experienced multiple PNES episodes upon entering their bathroom. Once bathroom access was restricted and the patient was supervised with a bedside commode, they experienced no further episodes on the unit. With no other potential triggers identified, we hypothesized that the patient’s symptoms were evoked by their reflection in the bathroom mirror, causing such great distress that it resulted in dissociation and PNES. This is a case of mirror-gazing potentially inducing PNES in a non-binary patient.

## Introduction

As its name suggests, psychogenic non-epileptic seizures (PNES) are functional seizures without epileptic brain wave abnormalities on an electroencephalogram (EEG) [1]. This condition's prevalence is approximately 100 per 100,000 persons within the United States [2]. Furthermore, up to 10% of patients thought to have benzodiazepine-refractory status epilepticus on multiple antiepileptic medications were found to have PNES [3]. Erroneous administration of antiepileptics can delay PNES diagnosis and worsen outcomes [4]. Thus, it is crucial for providers to distinguish epileptic seizures from PNES.

Although functional seizures are not due to an organic cause, they are genuine neurological symptoms, and are not feigned [5,6]. Additionally, the burden of PNES - including cognitive impairment, social consequences, and healthcare costs - is substantial and comparable in magnitude to epilepsy [7].

Transgender and gender non-conforming individuals have a higher prevalence of various neuropsychiatric disorders, including but not limited to functional seizures, major depressive disorder (MDD), and migraines [8,9]. Gender-affirming care for these individuals is associated with improvement or resolution of their functional neurological symptoms [10].

The neuropsychiatric pathology underlying PNES is not fully understood. One leading theory is that they are manifestations of a dissociative phenomenon, hence they are often referred to as dissociative seizures [11]. Dissociation is defined as a disruption of perception, identity, body representation, and motor control [5]. If such dissociative feelings are severe enough, they may potentially induce functional neurological disorders such as PNES. This is supported by literature showing that PNES and epilepsy patients both score higher on the Dissociative Experiences Scale (DES) compared to healthy controls [11,12].

Mirror-gazing, the act of intently staring at one’s self-reflection for a prolonged period, is one potential trigger for dissociation in patients with PNES [13]. However, research in this area remains limited, and there are currently no reports linking mirror-gazing with dissociative PNES in patients with gender dysphoria.

## Case presentation

A 16-year-old non-binary patient (pronouns they/them) presented to the emergency department (ED) following ingestion of rubbing alcohol with suicidal intent. Serum salicylate, acetaminophen, anion gap, and creatinine levels were within normal limits in the ED. The patient was in distress but was alert and oriented. Given their episodes of hematemesis, they were admitted to the hospitalist team for medical management and close monitoring.

The patient had a complex psychiatric history, including major depressive disorder (MDD), generalized anxiety disorder (GAD), post-traumatic stress disorder (PTSD), PNES, atypical anorexia nervosa, and attention-deficit/hyperactivity disorder (ADHD). There were no documented allergies or adverse reactions to medications. They were assigned as a biological female at birth and were reporting feelings of gender dysphoria for approximately four years. The patient reported that their relatives had been recently visiting from out of town and were not accepting of their gender identity. This, coupled with recently increasing feelings of underlying depression and distress, led the patient to their suicide attempt. History was obtained from the patient directly and corroborated from their parents and past medical records.

During their admission, the patient developed episodes of full-body seizures upon entering their bathroom. The patient’s EEG showed an initial increase in theta activity which normalized as the recording progressed. With otherwise no evidence of epileptiform activity on EEG, the episodes were attributed to PNES. Pharmacotherapy was not initiated, as antiepileptics can potentially worsen symptoms in PNES. Given the timeline of the patient’s symptoms and the absence of any other observable triggers, it was hypothesized that the patient’s functional seizures were elicited by their reflection in the bathroom mirror. The episodes occurred exclusively within the bathroom, and no other sources of reflection were available to the patient, such as smartphones or hand-mirrors. For safety, bathroom access was restricted and a bedside commode was provided. No further PNES episodes were observed during the remainder of the patient’s medical admission, further supporting our bathroom mirror-gazing hypothesis.

The patient had also been noted to develop new-onset gait disturbance as well as left upper extremity numbness. Similarly to their PNES, these symptoms were also most likely functional; however, unlike the PNES, these symptoms did not seem to be specifically triggered by bathroom entry. Laboratory workup including a vitamin B12 panel was unremarkable, and a thorough examination and assessment from the neurology team revealed no abnormalities consistent with a clear neurologic lesion or cause. Management was primarily supportive, the patient received supervised physical therapy (PT) daily to assist with ambulation and symptom improvement. The patient also received daily visitation from a hospital therapist, as well as multiple visits from inpatient therapy dogs and the psychiatry team. On this treatment plan, the patient saw an improvement in their functional neurological symptoms.

The hospitalist team managed the patient for esophagitis secondary to rubbing alcohol ingestion. This was achieved through bowel rest, proton pump inhibitors (PPIs), and intravenous (IV) hydration. Throughout their medical management, the patient continued to endorse persistent depression and suicidal ideation, necessitating one-on-one sitter supervision. Once medically cleared and stabilized, the patient was transferred to the inpatient psychiatry unit. Here, the patient began to gradually improve from a psychiatric stand-point. They continued to be closely supervised by a sitter for their suicidal thoughts and self-harming behaviors. Additionally, their sertraline was titrated up to 200 mg daily, and their nighttime trazodone was increased to 150 mg as needed. The patient also engaged in daily therapy sessions, including individual dialectical behavioral therapy (DBT), art therapy, and peer-to-peer group support.

The patient responded positively to these interventions, and their condition gradually improved. For two weeks prior to discharge, they consistently displayed safe behaviors without the need for a one-on-one sitter. Such accretion indicated the patient was stable for discharge, and they were transferred to a residential treatment center for adolescents.

## Discussion

This case highlights a novel potential trigger for PNES - mirror-gazing. While PNES has been associated with gender dysphoria, trauma, and psychological distress, there are no previous reports of visual self-triggers leading to functional seizures in transgender or non-binary patients.

**Figure 1:**
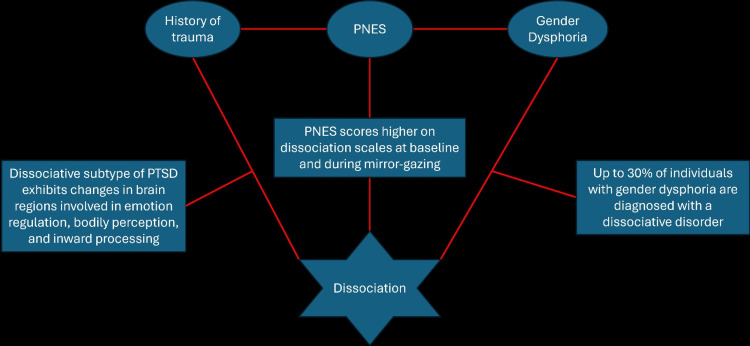
The relationship between PNES, gender dysphoria, trauma, and dissociation. Transgender and non-binary patients, particularly those with ongoing feelings of gender dysphoria, are at increased risk for PNES. Both PNES and gender dysphoria commonly copresent with a history of trauma and dissociative disorders. The neurobiological basis for these associations remains to be fully understood. PNES: psychogenic non-epileptic seizures; PTSD: post-traumatic stress disorder. The image is created by the author (Brendan Coyne) of this study.

Dissociation seems to be the unifying theme linking these processes together (Figure 1). It is increasingly recognized as a central mechanism underlying gender dysphoria and functional neurological disorders, including PNES. For instance, patients with PNES report stronger feelings of dissociation during mirror-gazing tests compared to healthy controls [13]. Additionally, approximately 30% of individuals with gender dysphoria experience concomitant dissociative disorders, which can be elicited by severe distress [14].

**Figure 2:**
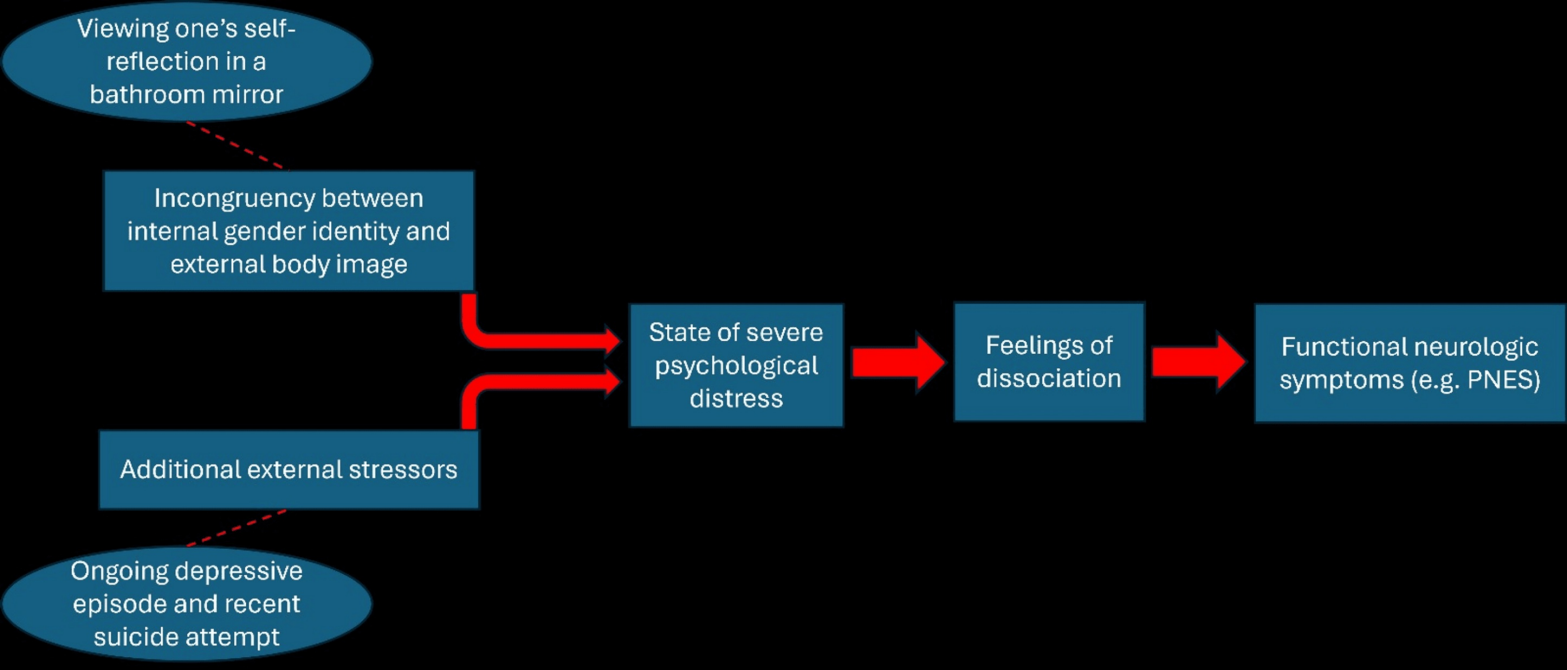
Summary of the proposed pathophysiology of PNES in patients with gender dysphoria. Ovals and dashed lines represent events that occurred in our patient that may have potentially contributed to their symptom development. Rectangular boxes and solid arrows represent pathological events that have been documented in the literature. PNES: psychogenic non-epileptic seizures The image is created by the author (Brendan Coyne) of this study.

Prior research has suggested that in individuals with gender dysphoria, visual stimuli that reinforce a discordance between internal gender identity and external appearance can provoke acute distress [15-17]. A similar process is observed in patients with body dysmorphic disorder and eating disorders [18]. It is possible that this distress can cause such an intense dissociation that it induces functional neurological symptoms, which may explain why our patient’s reflection in their bathroom mirror became a potent trigger for PNES episodes (Figure 2).

Neuroimaging studies support the link between dissociation and PNES, showing altered activity in brain regions involved in bodily perception, self-identity, and emotional regulation [19]. On functional magnetic resonance imaging (fMRI), abnormal signaling between structures like the basolateral amygdala and middle frontal gyrus is implicated in dissociative PTSD, which is commonly comorbid with PNES and gender dysphoria [19,20]. Hence, it is possible that such structures may play a role in PNES triggered by mirror-gazing in patients with gender dysphoria.

Much of this is theoretical, as there is a dearth of dedicated research in this area. This study aimed to address this gap by reporting the first known case of mirror-gazing as a potential trigger for PNES in a non-binary patient. However, there are limitations to this study. For instance, although we provided objective data to rule out epilepsy in this patient, we were unable to objectively measure the patient’s dissociation with the dissociative experiences scale. This was due to the prioritization of their medical stabilization at the time. Additionally, the individualized nature inherent to case reports limits the generalizability of the present study. We encourage providers to report any similar findings they may observe in future patients. Future research should also focus on cementing a neurobiological basis for the proposed mechanism of dissociation and PNES.

## Conclusions

We demonstrate the successful medical and psychiatric management of a suicidal non-binary patient with PNES. Both the patient’s psychiatric and functional neurological symptoms improved upon consistent therapeutic measures, including DBT and supportive therapy. Additionally, the patient’s PNES episodes ceased upon bathroom restriction. This case underscores the importance of assessing potential environmental and sensory triggers in patients with PNES, particularly those with gender dysphoria. Future research should further explore the role of visual self-perception in functional neurological disorders to improve therapeutic interventions.
